# Spermatozoa scattering by a microchannel feature: an elastohydrodynamic model

**DOI:** 10.1098/rsos.140475

**Published:** 2015-03-18

**Authors:** T. D. Montenegro-Johnson, H. Gadêlha, D. J. Smith

**Affiliations:** 1 Department of Applied Mathematics and Theoretical Physics, Centre for Mathematical Sciences, University of Cambridge, Wilberforce Road, Cambridge CB3 0WA, UK; 2 School of Mathematics, University of Birmingham, Edgbaston, Birmingham B15 2TT, UK; 3 Centre for Human Reproductive Science, Birmingham Women's NHS Foundation Trust, Mindelsohn Way, Edgbaston, Birmingham B15 2TG, UK; 4 Wolfson Centre for Mathematical Biology, University of Oxford, Mathematical Institute, Woodstock Road OX2 6GG, UK; 5 School of Engineering and Centre for Scientific Computing, University of Warwick, Coventry CV4 7AL, UK

**Keywords:** Stokesian swimming, fluid–structure interaction, human sperm

## Abstract

Sperm traverse their microenvironment through viscous fluid by propagating flagellar waves; the waveform emerges as a consequence of elastic structure, internal active moments and low Reynolds number fluid dynamics. Engineered microchannels have recently been proposed as a method of sorting and manipulating motile cells; the interaction of cells with these artificial environments therefore warrants investigation. A numerical method is presented for large-amplitude elastohydrodynamic interaction of active swimmers with domain features. This method is employed to examine hydrodynamic scattering by a model microchannel backstep feature. Scattering is shown to depend on backstep height and the relative strength of viscous and elastic forces in the flagellum. In a ‘high viscosity’ parameter regime corresponding to human sperm in cervical mucus analogue, this hydrodynamic contribution to scattering is comparable in magnitude to recent data on contact effects, being of the order of 5°–10°. Scattering can be positive or negative depending on the relative strength of viscous and elastic effects, emphasizing the importance of viscosity on the interaction of sperm with their microenvironment. The modulation of scattering angle by viscosity is associated with variations in flagellar asymmetry induced by the elastohydrodynamic interaction with the boundary feature.

## 2. Introduction

Human sperm propel themselves by propagating a travelling wave along a single, active flagellum; this motility is essential for migration through the female reproductive tract and natural fertilization. Recent work with microfluidic devices [1,2] has suggested the ability to direct and sort cells through their own motility, a potentially valuable advance in assisted reproduction therapy and in the livestock industry. Cell scattering at simple geometric features, such as the outside of a corner, appear to be dependent on viscosity and temperature; developing mechanical models to understand, interpret and optimize these effects for their exploitation is therefore of considerable interest. We will develop a mathematical model of a cell interacting with its environment, and its computational implementation, and study the dynamics of a realistic model sperm swimming over a backstep feature to study the effect of elastic, viscous and geometric parameters. The model will combine geometric nonlinearity of the elastic flagellum with non-local hydrodynamic interactions, and will be solved numerically via an implicit finite difference method for the elastohydrodynamic equations, combined with a hybrid slender body theory/boundary integral method for the low Reynolds number fluid dynamics.

The motor apparatus driving the flagellar waveform is a remarkably phylogenetically conserved structure known as the axoneme. The axoneme in human sperm comprises nine doublet microtubules, linked to each other and a central pair by passive elastic structures, with additional stiffening from outer dense fibres and the fibrous sheath (for a recent review focused on mechanically relevant features, see [3]). Motor proteins bound to the microtubules exert forces on adjacent doublets in a coordinated manner to induce bending moments along the length of the flagellum, causing bending, which is in turn resisted by the surrounding fluid. The fluid mediates interactions with surrounding surfaces and other cells; the flagellar waveform emerges from this nonlinear coupling.

Machin [4] showed that in order to generate experimentally observed waveforms the flagellum must actively bend along its length, and developed a linearized theory that has formed the basis of many subsequent studies. The theory that bending is produced by relative sliding of internal microtubules was subsequently proposed by Satir [5], and the sliding mechanism was modelled in early studies by Brokaw [6,7], using the formalism of an active internal moment per unit length in an elastic filament. The regulation of the active motor proteins that cause this sliding, and their oscillatory behaviour, is however a subject of continuing enquiry [8–10], with modelling playing an important role in comparing regulatory theories [11]. A number of studies since the 1970s have provided significant insights into how potential mechanisms of dynein regulation can produce the types of bending waves observed in nature (e.g. [8,12–15]).

The importance of large-amplitude elastohydrodynamic flagellar modelling was established by Gadêlha *et al.* [16], who delineated the range of validity of small-amplitude elastic theory and showed that for sufficiently high viscosity relative to flagellar stiffness, a buckling instability can give rise to waveform asymmetry without domain boundaries or asymmetric internal actuation. The numerical implementation of Gadêlha *et al.*'s study built on a model of passive flexible fibres in shear flow [17], although replacing the non-local hydrodynamics of the latter with a local drag-velocity law. The combination of three-dimensional, time-dependent flow, with the hydrodynamic interactions arising from fixed and moving boundaries, with active filament mechanics is computationally demanding; the majority of sperm models until the last decade made similar approximations for the fluid dynamics, or small-amplitude linearization of the flagellar wave.

Liron, Gueron and colleagues (e.g. [18,19]) modelled cilia arrays, taking both non-local fluid dynamics and geometric nonlinearity into account, building on earlier work by for example Lighthill [20] and Hines & Blum [13]. However this formalism, expressed in terms of bending angles rather than flagellar position, does not appear to have been generalized to a free-swimming cell with the associated boundary condition resulting from the presence of a head. More recent work using the finite-element and finite-volume methods and cluster computing has also been focused on cilia [21]; another successful recent approach is the regularized stokeslet method combined with a generalized immersed boundary method [22].

While the fluid dynamic interaction of sperm with plane boundaries has received significant attention since the work of Rothschild over 50 years ago [23], motivating a number of experimental and theoretical studies [24–27], the interaction of sperm with ‘non-trivial’ geometric obstacles involving angles and curves or complex interfaces is a subject of growing recent interest [28–31].

Denissenko *et al.* [1] showed how sperm scatter at a range of angles when encountering the outside of a corner in an artificial microchannel maze, and that the scattering angle is modulated by viscosity; Kantsler *et al.* [2] studied the effect of very close interactions of sperm and the biflagellate algae *Chlamydomonas* with these features. The geometric nature of the female reproductive tract is also highly convoluted, further motivating the need for models which can accommodate complex wall shapes. These studies suggest tantalizing opportunities to direct and sort motile sperm on passive microdevices, however a better understanding of the subtle nonlinear physics of how flagellated swimmers interact with geometric features must be developed; to aid with this understanding we will develop a mathematical and computational approach which accounts for elasticity, viscosity and their interaction, without the need for large-scale computational resources. To this end, we will bring together the active elastic formulation of Gadêlha *et al.* [16] with the Lighthill–Gueron–Liron (LGL) theorem [18] for non-local slender body theory and the boundary element [32] and regularized stokeslet methods [33,34] to capture the influence of a non-trivial nearby surface. We will use this approach to explore how sperm scatter near geometric features due to elastohydrodynamic interaction over hundreds of flagellar beats with a single computer core, and quantify how the balance of viscosity and elasticity modulates this effect via changes to the flagellar waveform.

## 3. Mathematical model

The mathematical model of a sperm interacting with a geometric feature will be derived from (i) the Stokes flow equations, with a non-local hydrodynamic model, and (ii) geometrically nonlinear elasticity for an internally actuated flagellum. We will first derive the equations for the two parts of problem, before describing (iii) the numerical implementation.

### 3.1 Hydrodynamics

At microscopic scales, fluid dynamics can be modelled by the incompressible Stokes flow equations

3.1 $$0=-\nabla p+\mu \nabla^{2}u,\;\nabla \cdot u=0,$$

where **u** is velocity, *p* is pressure and *μ* is dynamic viscosity. For our problem, these equations will be augmented with the no-slip, no-penetration condition **u**(**X**)=**X**_*t*_ for points **X** on the solid boundary, where subscript *t* denotes time derivative.

The linearity of the Stokes flow equations enables the construction of solutions to satisfy boundary conditions via discrete and/or continuous sums of suitably-weighted fundamental solutions. These techniques replace solid surfaces, such as the sperm flagellum, head and its surrounding microenvironment, by line or surface distributions of immersed forces. A concentrated point force located at **y** with strength **F** produces a velocity field (the ‘stokeslet’),

3.2 $$u_{j}(\text{x})=S_{jk}(\text{x},\text{y})F_{k},\;\text{where}\;S_{jk}(\text{x},\text{y})=\frac{1}{8\pi \mu }\left(\frac{\delta_{jk}}{|\text{x}-\text{y}|}+\frac{\left(x_{j}-y_{j})(x_{k}-y_{k}\right)}{|\text{x}-\text{y}{|}^{3}}\right),$$

the symbol *δ*_*jk*_ being the Kronecker delta tensor and the summation convention being used. The symbol **S**(***x***,***y***) will be used to denote the second rank tensor in equation (2.2). It will also be convenient to make use of the regularized stokeslet **S**^*ϵ*^ of Cortez [35], which corresponds to a spatially smoothed force; a frequently used implementation in three dimensions [33] takes the form

3.3 $$S_{jk}^{\epsilon }(\text{x},\text{y})=\frac{1}{8\pi \mu }\frac{\delta_{jk}(|\text{x}-\text{y}{|}^{2}+2\epsilon^{2})+(x_{j}-y_{j})(x_{k}-y_{k})}{{\left(|x-y{|}^{2}+\epsilon^{2}\right)}^{3/2}}.$$

The parameter *ϵ*>0 defines the length scale over which the point force is smoothed; this smoothness property is particularly convenient for the formulation of boundary integral methods.

The LGL theorem [19], an extension of the work of Lighthill [20], derives from a line distribution of singular stokeslets and source dipoles: an approximate expression for the flow field at the surface of a moving slender body, accurate to $O(\sqrt{b/L})$, where *b* is the radius and *L* is the flagellar length. Ignoring image systems, which are not required in our formulation, and using the properties of the stokeslet to reorder the source and field points, we have the expression for the approximate velocity field produced by the slender body **v**,

3.4 $$\begin{array}{rl} \text{v}(\text{X}(s_{0},t)) & =-\frac{1}{\xi_{∥}}({\text{f}}_{\mathrm{vis}}\cdot \hat{\text{s}})\hat{\text{s}}-\frac{1}{\xi_{⊥}}({\text{f}}_{\mathrm{vis}}\cdot \hat{\text{n}})\hat{\text{n}}-\frac{1}{\xi_{⊥}}({\text{f}}_{\mathrm{vis}}\cdot \hat{\text{b}})\hat{\text{b}}\\ & \;-\int_{|s-s_{0}|>q}\text{S}(X(s_{0},t),X(s,t))\cdot {\text{f}}_{\mathrm{vis}}(s,t)\;ds. \end{array}$$

Here and in what follows, 0≤*s*≤*L* is an arclength parametrization for the flagellum, and **f**_*vis*_ is the viscous force per unit length exerted by the fluid on the flagellum. The coefficients *ξ*_∥_ and *ξ*_⊥_ are parallel and perpendicular resistance coefficients similar to those of Gray & Hancock [36] and take the form

3.5 $$\xi_{⊥}=\frac{8\pi \mu }{1+2\ln(2q/b)},\;\xi_{∥}=\frac{8\pi \mu }{-2+4\ln(2q/b)}\;\mathrm{and}\;\gamma =\frac{\xi_{⊥}}{\xi_{∥}},$$

the parameter *q* being a length scale chosen intermediate in magnitude between *b* and *L*. The symbols $\hat{\text{s}}$, $\hat{\text{n}}$ and $\hat{b}$ are unit tangent, normal and binormal. Whereas Gueron and Liron [18,19] considered the dynamics of a cilium projecting from a plane boundary, and hence the associated image systems, in this study we will not require these terms because surfaces will be represented via boundary integrals.

Equation (2.4) can be considered a non-local extension of resistive force theories, which retain only the first three terms. To couple LGL to the elastohydrodynamic model of Gadêlha *et al.* [16], we will rewrite these terms in another commonly used form, $-(1/\xi_{⊥})(\text{I}+(\gamma -1)\hat{\text{s}}\hat{\text{s}})\cdot {\text{f}}_{\mathrm{vis}}$, with *γ*=*ξ*_⊥_/*ξ*_∥_ playing a similar role to the drag anisotropy ratio of resistive force theory, but depending on the choice of *q*. The precise value of *q* is not critical provided that *b*<*q*≪*L* because changes to the resistance coefficients are accompanied by changes to the integrals; for our study with *b*=0.01*L*, we choose *q*=0.1*L*, leading to *γ*≈1.4.

To model a sperm, we will consider a cell with a rigid head as well as a flagellum, swimming near a rigid step-like surface. The linearity of the Stokes flow equations means that a solution satisfying the additional no-slip boundary conditions associated with the head and the wall may be constructed by linear superposition. Moreover, the Lorentz reciprocal relation and its regularized analogue [33] enable the representation of these surfaces by boundary integrals; rigidity of the surfaces enables the use of single layer boundary integral representations [37], p. 32. In this study, we will use a hybrid approach, representing the head via a surface distribution of singular stokeslets with stress ***ϕ***^H^, discretized via BEMLIB [32], and the wall by regularized stokeslets and boundary elements, with stress ***ϕ***^*W*^ [33,34]. The full fluid dynamic model for the velocity field on the surface of the flagellum is therefore

3.6 $$\begin{array}{rl} \text{u}(\text{X}(s_{0},t)) & =-\frac{1}{\xi_{⊥}}(\text{I}+(\gamma -1)\hat{\text{s}}\hat{\text{s}})\cdot {\text{f}}_{\mathrm{vis}}-\int_{|s-s_{0}|>q}\text{S}(\text{X}(s_{0},t),\text{X}(s,t))\cdot {\text{f}}_{\mathrm{vis}}(s,t)\;ds\\ & \;-\iint_{H(t)}\text{S}(\text{X}(s_{0},t),\text{y})\cdot \phi^{H}(\text{y},t)\;dS_{\text{y}}-\iint_{W}{\text{S}}^{\epsilon }(\text{y},\text{X}(s_{0},t))\cdot \phi^{W}(\text{y},t)\;dS_{\text{y}}. \end{array}$$

Similar equations, but with the first two terms replaced by a single slender body integral $-\int_{0}^{L}\text{S}\cdot {\text{f}}_{\mathrm{vis}}\;ds$, hold on the surface of the head and the wall. In the next section, we will discuss the equations of an internally driven elastic flagellum, and their coupling to the fluid mechanics.

### 3.2 Elastohydrodynamics

The elastohydrodynamic formulation we will work with was derived by Tornberg & Shelley [17], and extended to an internally driven flagellum by Gadêlha *et al.* [16]; the central feature of this approach is to formulate the problem in terms of the flagellar position **X**(*s*,*t*) and line tension *T*(*s*,*t*). Alternative approaches based on bending angles and curvatures [38,39] have also been pursued, as has complex curvature [40]. The internal elastic contact force **F**_*int*_ and moment **M**_*int*_ exerted on the proximal flagellum [0,*s*_0_) by the distal flagellum (*s*_0_,*L*), respectively, are given by

3.7 $${\text{F}}_{\mathrm{int}}=-E{\text{X}}_{sss}+m\hat{\text{n}}+T{\text{X}}_{s}\;\mathrm{and}\;{\text{M}}_{\mathrm{int}}\wedge {\text{X}}_{s}=E{\text{X}}_{ss},$$

where *E* is constant elastic modulus and *m*(*s*,*t*) is a prescribed active moment density representing the internal flagellar motors. Balancing elastic and viscous forces acting on a segment of flagellum (*s*_0_,*s*_0_+*δs*) and taking the limit as δs→0 yields

3.8 $${\text{f}}_{\mathrm{vis}}+\partial_{s}(-E{\text{X}}_{sss}+m\hat{\text{n}}+T{\text{X}}_{s})=0.$$

Noting that $\hat{\text{s}}={\text{X}}_{s}$, the local term of equation (2.6) can then be written as

3.9 $$\begin{array}{rl} -\frac{1}{\xi_{⊥}}(\text{I}+(\gamma -1)\hat{\text{s}}\hat{\text{s}})\cdot {\text{f}}_{\mathrm{vis}} & =-E({\text{X}}_{ssss}+(\gamma -1)({\text{X}}_{s}\cdot {\text{X}}_{ssss}){\text{X}}_{s})+T{\text{X}}_{ss}+\gamma T_{s}{\text{X}}_{s}\\ & \;+m_{s}\hat{\text{n}}+\gamma m{\hat{\text{n}}}_{s}. \end{array}$$

For brevity, we will write the non-local (integral) velocities from equation (2.6) as **V** (written out explicitly in the appendix, equation (.2)). Non-dimensionalizing with scales *L* for position, 1/*ω* for time, *ωL* for velocity and *E*/*L*^2^ for tension and moment density yields the following dimensionless elastohydrodynamic equation:

3.10 $${\mathrm{Sp}}^{4}({\text{X}}_{t}-\text{V})=-{\text{X}}_{ssss}-(\gamma -1)({\text{X}}_{s}\cdot {\text{X}}_{ssss}){\text{X}}_{s}+T{\text{X}}_{ss}+\gamma T_{s}{\text{X}}_{s}+m_{s}\hat{\text{n}}+\gamma m{\hat{\text{n}}}_{s}.$$

The parameter Sp=*L*(*ξ*_⊥_*ω*/*E*)^1/4^ is the *sperm number*, which quantifies the relative importance of viscous and elastic effects. This model can be seen as an extension of linear models (such as Camalet *et al.* [14]) by the inclusion of the nonlinear terms on the right-hand side, and an extension of hydrodynamically local models (such as Gadêlha *et al.* [16]) by the inclusion of the **V** term on the left-hand side.

Similar to Gadêlha *et al.* [16], the inextensibility constraint ∂_*t*_(**X**_s_⋅**X**_s_)=0 can be used with the elastohydrodynamic equation (2.10) to deduce an ordinary differential equation which must be satisfied by the line tension *T*,

3.11 $$\begin{array}{rl} -{\mathrm{Sp}}^{4}{\text{V}}_{s}\cdot {\text{X}}_{s} & =\gamma T_{ss}-{\text{X}}_{ss}\cdot {\text{X}}_{ss}T+3\gamma {\text{X}}_{sss}\cdot {\text{X}}_{sss}+(1+3\gamma ){\text{X}}_{ss}\cdot {\text{X}}_{ssss}\\ & \;+(\gamma +1)m_{s}{\hat{\text{n}}}_{s}\cdot {\text{X}}_{s}+m{\hat{\text{n}}}_{ss}\cdot {\text{X}}_{s}. \end{array}$$

The above equation is derived via the identity 3**X**_*ss*_⋅**X**_*sss*_+**X**_s_⋅**X**_*ssss*_=0 and its derivative with respect to *s*. As previously [17,16], we introduce the term *λSp*^4^(1−**X**_s_⋅**X**_s_) to the left-hand side of equation (2.11) to dampen numerical errors in flagellar length. The value used in this study is *λ*=80, though as found by Gadêlha *et al.* the solution is insensitive to the precise value of *λ*.

The final part of the mathematical model is the specification of the boundary conditions for equations (2.10) and (2.11). The assumption of zero contact force and moment at the distal (*s*=1) tip of the flagellum combined with the elasticity equations (2.7) yield (in dimensionless variables)

3.12 $$0=-{\text{X}}_{sss}+m\hat{\text{n}}+T{\text{X}}_{s}\;\mathrm{and}\;0={\text{X}}_{ss}\;\text{at}\;s=1.$$

Taking the dot product of the first equation with **X**_s_, using the identity **X**_s_⋅**X**_*sss*_=−**X**_*ss*_⋅**X**_*ss*_ and the second equation yields the distal tension boundary condition, *T*=0.

At the proximal end of the flagellum, the boundary conditions are given by considering the force and moment exerted by the fluid on the head. We denote these quantities **F**^H^ and **M**^H^ and non-dimensionalize them with the elastic scalings *E*/*L*^2^ and *E*/*L*, respectively. In the inertialess Stokes flow regime, the total force and moment acting on the head are zero, so by Newton's third law, the force and moment on the flagellum at *s*=0 are also given by **F**^H^ and **M**^H^, respectively. With the appropriate scalings, the proximal boundary conditions are then

3.13 $${\text{F}}^{H}={\text{X}}_{sss}-m\hat{\text{n}}-T{\text{X}}_{s}\;\text{and}\;{\text{M}}^{H}\wedge {\text{X}}_{s}=-{\text{X}}_{ss}+M\hat{\text{n}},\;\text{at}\;s=0,$$

where $M=\int_{0}^{1}m\;ds$. From these equations, we also derive the tension condition at the proximal end, ${\text{F}}^{H}\cdot {\text{X}}_{s}=-{\text{X}}_{ss}\cdot {\text{X}}_{ss}-T$. The calculation of the quantities **F**^H^ and **M**^**H**^ with non-local hydrodynamic interaction is described in more detail in the next section and the appendix. Finally, we introduce the translational and angular velocity **U**^H^ and ***Ω***^H^ of the head; while **U**^H^ and two components of the angular velocity are constrained by knowledge of the function **X**, there is an independent rotational component of the motion that defines the principal bending plane of the flagellum. These quantities will be determined by kinematic considerations and the implementation of the boundary conditions.

To complete the mathematical model, it is necessary to specify the internal active moment *m*(*s*,*t*). Gadêlha *et al.* [16] used travelling waves of internal moment, which calculations from experiment [3] confirm are a good model. We therefore specify in dimensionless units, $m(s,t)=m_{0}\cos(ks-t)$.

### 3.3 Numerical implementation

The elastohydrodynamic equation (2.10) is treated with a Crank–Nicolson-type finite difference discretization, with the second-order central differences in the interior, and third-order one-sided difference for the boundary conditions, using coefficients taken from Fornberg [41]. The higher order boundary stencil produced comparable errors to the central stencil on polynomial test functions. Both linear and nonlinear terms are treated implicitly; nonlinearity of these equations is dealt with by performing an iterative process on every time step, with the operator on the left-hand side at *t*+*dt* being linearized as

3.14 $$-{\text{X}}_{ssss}-(\gamma -1)({\tilde{\text{X}}}_{s}\cdot {\text{X}}_{ssss}){\tilde{\text{X}}}_{s}+T{\tilde{\text{X}}}_{ss}+\gamma T_{s}{\tilde{\text{X}}}_{s}+f_{s}\tilde{\text{n}}+\gamma f{\tilde{\text{n}}}_{s},$$

variables with tildes denoting that values from the previous iteration are taken.

The non-local hydrodynamic term **V** in equation (2.10) is approximated by forming the slender body/boundary integral problem of determining **f**_*vis*_, ***ϕ***^H^ and ***ϕ***^*W*^ using the most recent approximations to $\tilde{\text{X}}$ and ${\tilde{\text{X}}}_{t}$ available; details are given in the appendix.

At the first iteration of each time step, the converged values from the previous time step are used as starting guesses for all variables, except for **X** which is approximated via linear extrapolation. The nonlinear iteration is terminated when the maximum difference in position between successive iterations relative to the distance travelled by the flagellum over the time step falls below 0.5%. Similarly, the auxiliary equation for the tension at *t*+*dt* is linearized as

3.15 $$\begin{array}{rl} {\mathrm{Sp}}^{4}(\lambda (1-{\tilde{\text{X}}}_{s}\cdot {\text{X}}_{s})-{\tilde{\text{V}}}_{s}\cdot {\tilde{\text{X}}}_{s}) & =\gamma T_{ss}-({\tilde{\text{X}}}_{ss}\cdot {\tilde{\text{X}}}_{ss})T+3\gamma {\tilde{\text{X}}}_{sss}\cdot {\text{X}}_{sss}\\ & \;+(1+3\gamma ){\tilde{\text{X}}}_{ss}\cdot {\text{X}}_{ssss}+(\gamma +1)({\tilde{\text{n}}}_{s}\cdot {\tilde{\text{X}}}_{s})m_{s}\\ & \;+({\tilde{\text{n}}}_{ss}\cdot {\tilde{\text{X}}}_{s})m. \end{array}$$

Each iteration requires the solution of a linear system for the unknown discrete values of **X**(*s*_*l*_,*t*_*n*+1_), *T*(*s*_*l*_,*t*_*n*+1_), **U**^H^ and ***Ω***^H^, where *l*=0,…,*N*_s_ denotes the spatial grid coordinate and *n*=0,1,… the time step. We found that *N*_s_=160 and 200 time steps per beat were sufficient to yield accurate results. The discrete form of equations (2.10) and (2.11) provide 4(*N*_s_+1)+6=650 linear equations, the additional six equations arising from the translational and angular velocity of the cell head. The nonlinear correction is then a system of 3(*N*_s_+*N*_h_+*N*_b_) linear equations, where *N*_h_ and *N*_b_ are the number of elements on the head and domain boundary, respectively.

To implement the boundary conditions (2.12) and (2.13), the force and moment on the head are *a priori* unknown and need to be determined as part of the coupled problem. The force and moment are decomposed into a linear part, given by the grand resistance matrix associated with rigid body motion in the vicinity of the wall, and an additional subleading correction resulting from the influence of the flagellum. Following non-dimensionalization with the elasticity scalings, the force and moment on the head may then be expressed as

3.16 $$\left(\begin{array}{c} {\text{F}}^{H}\\ {\text{M}}^{H} \end{array}\right)={\mathrm{Sp}}^{4}\left(\frac{\mu }{\xi_{⊥}}\right)R\cdot \left(\begin{array}{c} {\text{U}}^{H}\\ \Omega^{H} \end{array}\right)+\left(\begin{array}{c} \Delta {\text{F}}^{H}\\ \Delta {\text{M}}^{H} \end{array}\right),$$

where Δ**F**^H^, Δ**M**^H^ are corrections for the effect of the flagellum. The calculations of R and the corrections are described in the appendix.

In summary, each time step requires a number of iterations to solve the nonlinear problem and each iteration involves the solution of a sparse linear system arising from the finite difference discretization of the elastohydrodynamic equations. The ‘right-hand side’ terms arising from the non-local hydrodynamic correction **V** and the non-local corrections to the force and moment balance Δ**F**^H^, Δ***Ω***^H^ require the solution of a slender body theory–boundary integral hydrodynamic problem. Calculation of the grand resistance matrix R requires the separate solution of a boundary integral problem with multiple right-hand sides to determine the force and moment resistances associated with the rigid body modes of the head and the wall interaction. The code is implemented in Fortran 90 (gfortran, GNU Compiler Collection); linear systems are equilibriated and solved by LU factorization with the LAPACK routines dgeequ and dgesv, respectively, and the boundary integrals over the sperm head are calculated with routines from BEMLIB [32]. A typical run of 200 beats with 500 boundary elements required approximately 24 h walltime on a single core of a 2.2 GHz Intel Sandy Bridge E5-2660 node.

## 4. Results

**Figure 1.**
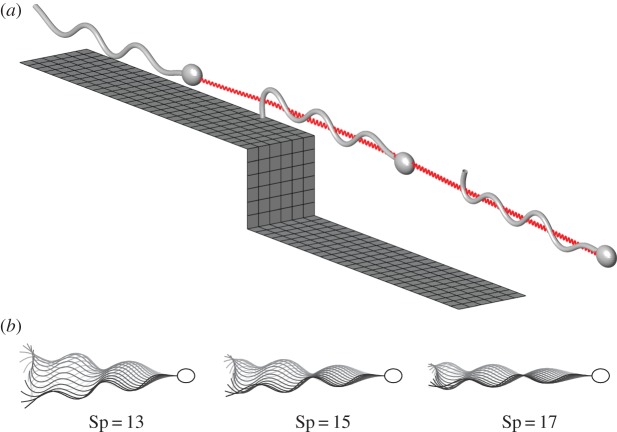
Example results from non-local elastohydrodynamic simulation. (*a*) Plot of the problem domain, including the boundary element meshes for the wall and swimmer, and a plot of the trajectory, computed at Sp=13. (*b*) Waveforms in infinite fluid of flagella driven by the same internal force at different sperm numbers. The effect of increasing sperm number is to reduce cell yaw and bending in the proximal end of the flagellum, as is observed in the waveform of sperm in high viscosity medium [42].

The numerical scheme is applied to predict the trajectory of a sperm-like cell swimming in an unbounded fluid at varying Sp, over a ‘backstep’ (the latter being shown in figure 1*a*), the limiting case of zero backstep height being referred to as a ‘strip’. As in Gadêlha *et al.* [16], we consider planar waveform actuation, which is appropriate for cells swimming through high viscosity fluids such as cervical mucus [42]. The semi-axes of the ellipsoidal head, modelled with the boundary element method, are *a*_*x*_=0.05*L*, *a*_*y*_=0.03*L*, *a*_*z*_=0.04*L*, which correspond to a 5×3×4 μm head for a flagellum of length *L*=50 μm. The swimmer is initially at rest, with a straight flagellum, and a ‘soft start’ is applied whereby the internal shear moment is initially low and smoothly increases to its maximum, reaching 99% after five beats. The sperm number of a human gamete can be approximated by using bending stiffness *E*≈5×10^−21^ Nm^2^, beat frequency 10–20 *Hz* giving an angular frequency *ω*≈100 rad s^−1^ [3]. Taking a flagellar radius of 0.5 μm, viscosity *μ*≈0.14 Pa⋅s (similar to mucus analogue [42]) yields the normal resistance coefficient *ξ*_⊥_≈0.503 and sperm number Sp≈15.8. Therefore, we will consider a range of sperm numbers between 13 and 17, fixing the magnitude of the internally generated shear-force *m*_0_=240 and wavenumber *k*=6*π*. The resulting waveforms are shown in figure 1*a*. As sperm number increases, beat amplitude is suppressed, as is observed for sperm in high viscosity medium [42], leading to a reduction in side-to-side yaw. All simulations in infinite fluid, i.e. with no nearby boundaries, produced trajectories which were straight overall, once the within-beat yaw was accounted for (data submitted to Dryad repository [43]); flagellar waveforms for Sp=13, 15 and 17 are shown in figure 1*b*,*c*.

**Figure 2.**
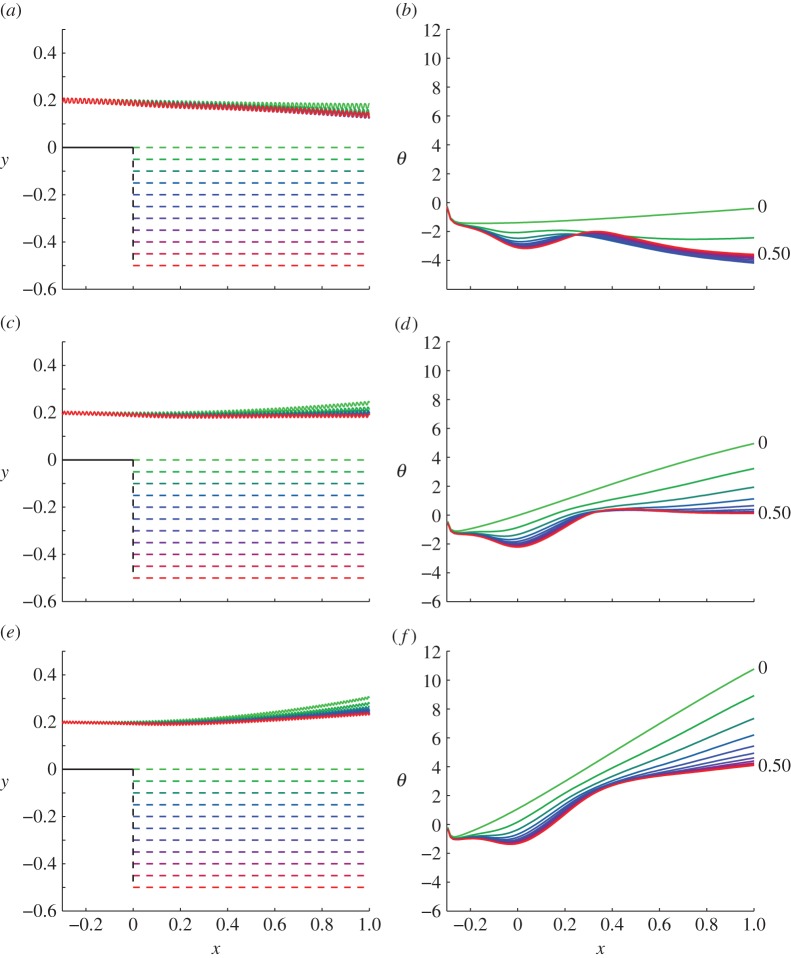
Projected trajectories (*X*(0,*t*),*Y* (0,*t*)) and angles of trajectories θ:=arctan dY/dX(s=0) for different sperm numbers as a function of changing the height of the backstep. (*a*,*b*) Sp=13, (*c*,*d*) Sp=15, (*e*,*f*) Sp=17. Colour corresponds to the backstep heights shown in (*a*,*c*,*e*).

Figure 2 shows a planar projection of the trajectories (*X*(0,*t*),*Y* (0,*t*)) and the tangent angle θ:=arctan⁡(dY/dX(s=0)) (in degrees) of those trajectories, of cells swimming over backsteps of varying height. The derivative d*Y*/d*X* is calculated numerically by sampling the trajectory at the temporal midpoint of each beat-cycle and taking centred differences. Colour indicates the trajectory over the backstep of the height in figure 2*a*,*c*,*e* with green denoting *h*=0 and red denoting *h*=0.5. Simulations were performed over backsteps of height *h*=0.05,0.1,…,0.5 and are displayed up to the time at which *X*(0,*t*)≥1.

The results in figure 2*a*,*c*,*e* suggest that the backstep affects swimmers at different sperm numbers differently, producing a range of scattering angles. However, it is important in these results to factor out the effects of the strip from the backstep. Taking the (lightest) green trajectory, representing a strip, as a baseline comparison, it is evident that for all sperm numbers the hydrodynamic effect of the backstep is to deflect the swimmer downwards relative to a strip trajectory. Figure 2*b*,*d*,*f* reveals that this downward deflection is not smooth, rather there is a sharp bump at *x*=0 where the head initially passes over the backstep, and a further bump at around *x*=0.3 where the effect of the step itself becomes subleading relative to boundary interactions between the head and the lower wall.

Simulations were also performed comparing the effect of the backstep to a ‘cliff’ geometry, with the lower portion of the backstep missing (data submitted [43]). After passing the backstep, cells swam straight as though in an infinite fluid, suggesting that the majority of the angular deflection occurs due to interaction with the lower boundary; boundary forces change suddenly over a step jump, and the cell acts as though it were above a higher boundary. Additionally, simulations over a strip at Sp=13 for different starting heights (data submitted [43]) showed that attraction to the surface initially increased and then decreased as height above the surface increased, which suggests that hydrodynamic boundary attraction is responsible for the behaviour in figure 2*a*,*b*.

**Figure 3.**
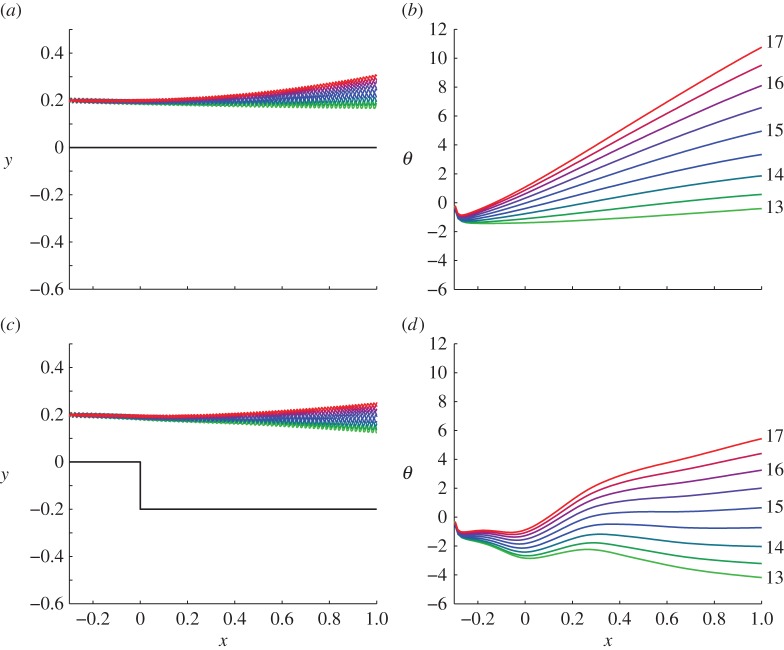
Projected trajectories (*X*(0,*t*),*Y* (0,*t*)) and angles of trajectories θ:=arctan dY/dX(s=0) for varying sperm number over fixed geometry. Panels (*a*,*b*) show trajectories and angles with a ‘strip’, i.e. zero backstep height, (*c*,*d*) with backstep height 0.2*L*. Colour is matched to sperm number, light green denoting Sp=13 and red denoting Sp=17, intermediate colours moving in increments of 0.5.

**Figure 4.**
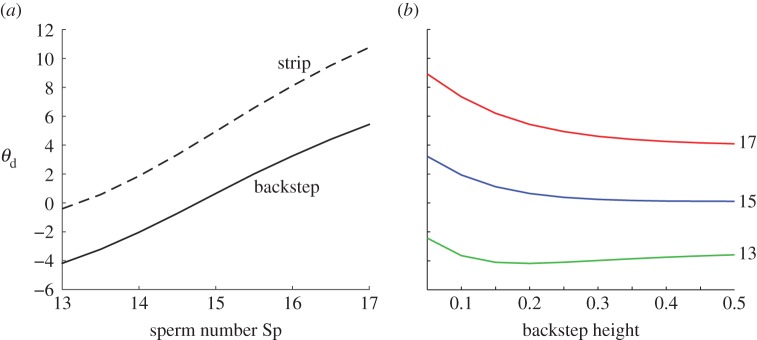
The effect of the backstep on scattering, showing ‘final’ deflection at *x*=1.0*L*, for (*a*) strip and backstep (*h*=0.2*L*) and a range of sperm numbers, (*b*) Sp=13,15,17 and a range of backstep heights. Colour in (*b*) denotes sperm number as indicated by labels on the right-hand side.

Figure 3 shows the effect of varying sperm number over finer increments for backstep height zero (*a*,*b*) and *h*=0.2*L* (*c*,*d*), with results summarized in figure 4*a*. Simulations were performed for Sp=13,13.5,…,17 over both a strip geometry and a backstep of height *h*=0.2*L*, so that the sperm cells initially start 0.2*L* above the surface, and then increase to around 0.4*L* after the backstep. In figure 3*a*–*d*, colour is matched to increasing sperm number, so that light green corresponds to Sp=13 and red to Sp=17. Figure 3*a*,*b* shows for a sperm swimming over a strip, the boundary repels the swimmer more at this close distance as sperm number is increased. This effect is to be expected because increasing the sperm number increases the relative strength of viscous to elastic forces, thus the effect of the boundary is likely to be enhanced as Sp increases. The initial dip in figure 3*b* is an artefact of the numerical soft start of our system, as the waveform emerges from a straight initial state.

Figure 3*c*,*d* shows a larger range of scattering angles than for fixed sperm number over various backstep heights, of the order of 10°. Furthermore, additional simulations (data submitted [43]) showed that this hydrodynamic deflection was not sensitive to the phase of the waveform as it passed over the backstep, in contrast to scattering due to contact forces (R. Goldstein 2014, personal communication). Figure 4*a* shows the effects of changing sperm number, giving the deflection for a strip, a backstep, and their difference. A slight increase in the magnitude of this difference is observed as sperm number is increased, owing to increased hydrodynamic interaction mediated by viscosity.

Figure 4*b* summarizes the effect of varying both backstep height and sperm number simultaneously, quantified by the ‘final deflection angle’ *θ*_d_, i.e. the value of *θ* for which *X*=*L*. At Sp=13 deflection is always negative, whereas for Sp=15, 17 deflection is always positive. The relationship between *θ*_d_ and *h* is non-monotone at the lower sperm number but is monotonic in the higher range. At Sp=13, the deflection angle initially increases in magnitude, then decreases after the maximum at around *h*=0.15*L*. This riser height corresponds to a distance of 0.35*L* between the cell and the boundary, which is where boundary attraction is strongest at this sperm number. For Sp=15, 17, the deflection angle decreases monotonically with backstep height in the range we have considered. This effect probably occurs because at these sperm numbers the strip causes the cell to pitch away. However in all cases, increasing backstep height to 0.5*L* results in a plateau.

**Figure 5.**
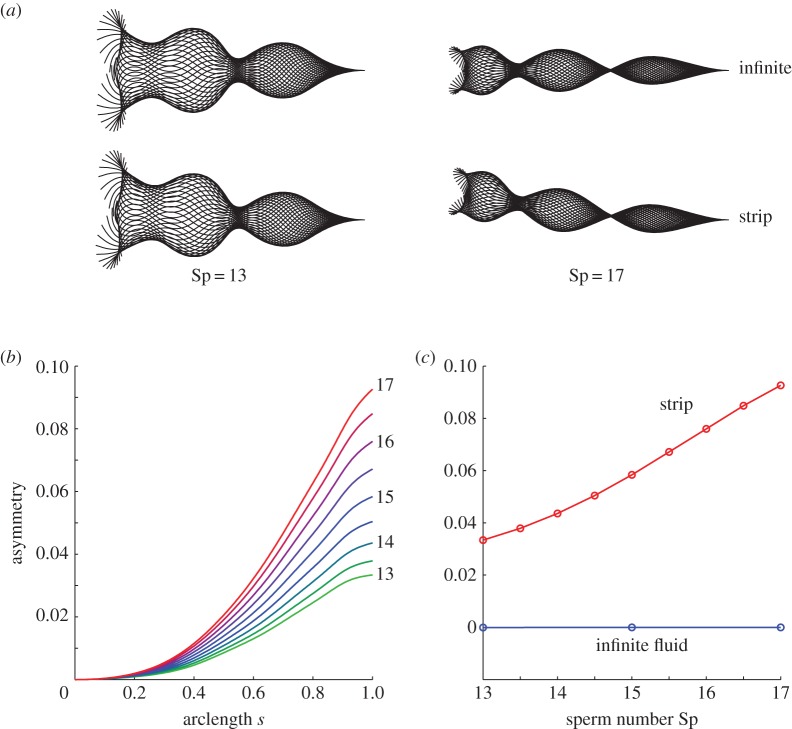
Boundary-induced waveform asymmetry, increasing with sperm number. (*a*) Symmetric waveforms of sperm flagella swimming in infinite fluid, and asymmetric forms over the boundary strip for Sp=13 and Sp=17. (*b*) Flagellar asymmetry (defined in text) as a function of arclength, for a cell swimming over a strip along the flagellum. Colour in (*b*) is matched to sperm number, light green denoting Sp=13 and red denoting Sp=17, intermediate colours moving in increments of 0.5. (*c*) Asymmetry at *s*=1 at the endpoint of the flagellum, as a function of sperm number Sp, showing an increase in asymmetry with sperm number for the strip (red), and negligible asymmetry for the infinite fluid case (blue).

The effects of the backstep on the waveform are summarized in figure 5, which show the waveform shape with and without the boundary, and quantitative measures of the asymmetry of the waveform. Recall that the flagellar actuation is symmetric; waveform asymmetry is produced due to increased hydrodynamic drag arising from proximity to the wall [44] affecting closer portions of the flagellum more than further portions. Figure 5*a* shows waveforms at sperm number Sp=13, 17 in infinite fluid as well as over a strip. In infinite fluid, the waveform is symmetrical for all sperm numbers considered, while the presence of a boundary gives rise to a waveform asymmetry that increases with Sp.

‘Asymmetry’ is quantified by sampling the flagellar wave every 41 numerical time steps (relative to a beat cycle of 200 time steps), projecting into the body frame and calculating the average lateral position relative to the body frame centreline over a fixed period, in this case beats 82–90. This quantity is plotted as a function of arclength in figure 5*b*; its distal (*s*=1) value is plotted in figure 5*c*.

Figure 5*b* plots asymmetry versus arclength for sperm numbers in the range 13–17, the effect being largest at higher sperm number. The asymmetry at the tip of the flagellum for a strip versus no boundary is shown in figure 5*c* as a function of sperm number.

## 5. Discussion

A numerical method for simulating the swimming of monoflagellate cells over geometric features was presented and applied to model sperm interacting with microchannel backstep feature. The scheme incorporates non-local hydrodynamics with large-amplitude active filament mechanics. We believe this method to be the simplest generalization of previous work that is capable of taking into account non-local hydrodynamic interaction geometrical features. The linearity of the Stokes flow equations entails that the largest error in our method arises from the LGL slender body theory, which is at worst on the order of the square root of the slenderness ratio. Accuracy of the method of regularized stokeslets is on the order of the regularization parameter near the boundary, and its square far from the boundary where the swimmer is located. Future work may consider boundary integral modelling of the flagellum also; however, we do not expect that this would qualitatively change swimmer trajectories.

The interaction between the cell and the lower boundary involves the competing effects of asymmetric hydrodynamic forces leading to waveform asymmetry and boundary repulsion, and the pitching behaviour associated with swimmer/boundary interaction [26]. At lower sperm number and at greater distances from the boundary, waveform asymmetry is smaller, and the cell pitches towards the boundary. At higher sperm number and closer distances from the boundary, waveform asymmetry is larger and the cell pitches away. The effect of the backstep is a sudden drop in the lower boundary, which changes the relative importance of these effects; waveform asymmetry is reduced relative to hydrodynamic attraction, and the net result is a deflection towards the lower boundary after the backstep relative to the expected trajectory over a strip (figure 2).

Analysing sperm scattering over a backstep, we found that hydrodynamic effects may be comparable in magnitude in the relatively high viscosity range considered to the contact interactions found experimentally by Kantsler *et al.* [2]. A transition is predicted from scattering towards the backstep at lower viscosity to scattering away from the backstep at higher viscosity. Qualitatively this behaviour is similar to the temperature-related transition in Kantsler *et al.*'s observations (with lower temperature corresponding to higher viscosity); the correspondence is not exact however, with Kantsler *et al.*'s observations being carried out with bull sperm in low viscosity buffer, and with cells exhibiting very close interaction with the boundary, compared with our longer range interactions and sperm number representative of human cells in mucus analogue that we chose to focus on in this study. Clearly integrating both surface interactions and hydrodynamics will be necessary to develop a comprehensive model, particularly at higher sperm number/viscosity.

The role of hydrodynamic interactions in determining surface attraction and more complex effects associated with boundary features continues to receive significant theoretical attention and is stimulating novel mathematical approaches [28–31,45]. Viscous interactions of course become increasingly important in high viscosity fluids such as mucus and laboratory analogues. Kantsler *et al.* [2] noted the need to take both elastic and steric interactions into account; modelling very short length scale or contact interactions, with either glass, epithelium, cumulus or even ciliated surfaces, and their effect on the flagellar wave, is a topic of importance, though numerical simulation requires taking account of the rapidly varying hydrodynamic force and electrostatic interactions as the swimmer approaches these boundaries. We hope that the numerically implicit method, potentially also combined with adaptive refinement of the boundary element meshes, will enable accurately resolved simulation of sperm-like swimmers in very near surface-contact in future work. Other valuable methods for modelling three-dimensional sperm motility and elastic-fluid interaction include models based exclusively on regularized stokeslets [46,47] and techniques such as stochastic rotation dynamics [48].

While we have used our model to examine a swimmer representative of human sperm, the approach is applicable to a much wider range of eukaryotic cells, including the sperm of other species and, with a slight reworking of the head boundary condition, biflagellate organisms such as the green alga *Chlamydomonas*. These species are of particular interest as they have been used as models for flagellar synchronization [49] and are relevant to energy-producing bioreactors [50]. For these systems, the model may also be extended to include a non-local hydrodynamic contribution from other swimmers. Larger swimming organisms, such as *Caenorhabditis elegans*, have also been shown to be significantly affected by interactions with a structured microenvironment [51,52].

Another application is the design and optimization of biomimetic artificial microswimmers (e.g. [53,54]). Because the model includes internal periodic actuation via prescribed bending moments, it might be used to optimize actuation for various purposes such as forward progress, subject to constraints such as fixed mechanical energy. Furthermore, the inclusion of geometrical boundary features and the use of sperm number allow such optimization to be tailored to specific environments. The elastohydrodynamic model can additionally be used to solve the inverse problem of estimating internal moments from observed flagellar data, potentially allowing us to examine how nature has optimized swimming in various environments and informing truly biomimetic design.

Despite the linearity of the Stokes flow equations, the interaction of sperm with their microenvironment presents a subtle nonlinear mechanics problem. Sperm scattering depends nonlinearly on the ratio between viscous and elastic forces, with even a simple backstep feature producing attractive or repulsive scattering of cells depending on parameter values. These scattering effects may be valuable in sorting cells in microdevices, in addition to giving insight into the complexity of how sperm interact with their microenvironment. The combination of mechanical models and experiment will provide the best way to understand and exploit these effects for biomedical applications.

## Data accessibility

Trajectory and waveform data supporting the findings in this paper have been submitted to Dryad; see reference [43].

## Appendix A. Calculation of hydrodynamic terms

Following non-dimensionalization, the hydrodynamic model yields the following equation for the dimensionless fluid velocity away from the flagellum:

A 1 $$\begin{array}{rl} {\mathrm{Sp}}^{4}U(x) & =\frac{\xi_{⊥}}{\mu }(\int_{0}^{1}\text{S}(x,X(s,t))\cdot f_{\mathrm{vis}}(s,t)\;ds+\iint_{H(t)}\text{S}(x,Y)\cdot \phi^{H}(Y,t)\;dS_{Y}\\ & \;+\iint_{W}{\text{S}}^{\epsilon }(x,Y)\cdot \phi^{W}(Y,t)\;dS_{Y}). \end{array}$$

The non-local contribution to the velocity **V** on the slender body is similarly given by

A 2 $$\begin{array}{rl} {\mathrm{Sp}}^{4}\text{V}(\text{X}(s_{0},t)) & =\frac{\xi_{⊥}}{\mu }(\int_{|s-s_{0}|>q}\text{S}(\text{X}(s_{0},t),\text{X}(s,t))\cdot {\text{f}}_{\mathrm{vis}}(s,t)\;ds\\ & \;+\iint_{H(t)}\text{S}(\text{X}(s_{0},t),\text{Y})\cdot \phi^{H}(\text{Y},t)\;dS_{\text{Y}}\\ & \;+\iint_{W}{\text{S}}^{\epsilon }(\text{X}(s_{0},t),\text{Y})\cdot \phi^{W}(\text{Y},t)\;dS_{\text{Y}}). \end{array}$$

At each step of the iterative solution to the nonlinear problem, the collocation code solves the integral equation

A 3 $${\mathrm{Sp}}^{4}\left(\begin{array}{c} {\text{X}}_{t}\\ {\text{U}}^{H}+\Omega^{H}\wedge ({\text{Y}}^{H}-{\text{X}}^{c})\\ 0 \end{array}\right)=\left(\begin{array}{c} -(\text{I}+(\gamma -1)\hat{\text{s}}\hat{\text{s}})\cdot {\text{f}}_{\mathrm{vis}}+{\mathrm{Sp}}^{4}\text{V}[{\text{f}}_{\mathrm{vis}},\phi^{H},\phi^{W}](\text{X})\\ {\mathrm{Sp}}^{4}\text{U}[{\text{f}}_{\mathrm{vis}},\phi^{H},\phi^{W}]({\text{Y}}^{H})\\ {\mathrm{Sp}}^{4}\text{U}[{\text{f}}_{\mathrm{vis}},\phi^{H},\phi^{W}]({\text{Y}}^{W}) \end{array}\right),$$

for the unknown hydrodynamic force per unit length **f**_*vis*_ and unknown stresses ***ϕ***^H^, ***ϕ***^*W*^.

The collocation code discretizes the flagellum with 160 elements, with the non-local contribution to the LGL slender body theory computed by the midpoint rule with constant force per unit length over each element. The force per unit area on the ellipsoidal head of 32 mesh elements is calculated using routines from BEMLIB [32] with 20 point Gauss–Legendre quadrature as described in detail in the appendix. The wall boundary is discretized into elements of width 0.075*L*, using regularized stokeslets with *ϵ*=0.01*L*. Integration is performed with repeated Gauss–Legendre quadrature with 4×4 points per element for the near-singular wall integrals, and a 2×2 point rule elsewhere.

To implement the boundary conditions (2.12) and (2.13), Gadêlha *et al.* [16] approximated the force and moment on the head by a grand resistance matrix [32] multiplying the velocity and angular velocity. In dimensional variables, the grand resistance matrix expresses the force and moment on a moving rigid body as

$$\left(\begin{array}{c} \text{F}\\ M \end{array}\right)=R\cdot \left(\begin{array}{c} \text{U}\\ \Omega \end{array}\right),\;\text{where}\;R=\left(\begin{array}{c} R^{F}\\ R^{M} \end{array}\right).$$

The blocks $R^{F}$ and $R^{M}$ are 3×6 matrices yielding the force and moment terms, respectively. For example, a sphere of radius *a* in the absence of hydrodynamic interactions would have dimensionless grand resistance matrix given by

$$R^{F}=(-6\pi \mu a\text{I}\;0)\;\mathrm{and}\;R^{M}=(0\;-8\pi \mu a^{3}\text{I}).$$

This approach is convenient because the linearity of the relationship means that the head velocity **U**^H^ and angular velocity ***Ω***^H^ can be dealt within the implicit formulation as unknowns in the linear algebra problem. To generalize to a non-local hydrodynamic model taking into account the effect of the flagellum and nearby boundary, the force and moment will be decomposed as consisting of part which is linear in velocity and angular velocity via resistance matrices and a remaining contribution from the flagellum. The matrices $R^{F}$ and $R^{M}$ are determined via the boundary integral method, taking into account the potentially highly significant effect of the wall feature, but not the subleading effect of the flagellum, which is accounted for as a correction, as described below.

Elastic scalings are used to non-dimensionalize all forces and moments, i.e. *E*/*L*^2^, *E*/*L* for **F**^H^ and **M**^H^, respectively, with *E*/*L*^3^ for force per unit length **f**_*vis*_ and *E*/*L*^4^ for stress ***ϕ***^H^, ***ϕ***^*W*^. The additional corrections Δ**F**^H^ and Δ***M***^H^ referred to in equation (2.16) are determined as part of the iterative process by performing a slender body/boundary integral calculation of ${\tilde{\text{f}}}_{\mathrm{vis}}$, ${\tilde{\phi }}^{H}$ and ${\tilde{\phi }}^{W}$ with the most recent approximation to $\tilde{\text{X}}$ available, yielding in dimensionless variables

A 5 $${\tilde{\text{F}}}^{H}=\iint_{H(t)}{\tilde{\phi }}^{H}\;dS\;\mathrm{and}\;{\tilde{\text{M}}}^{H}=\iint_{H(t)}(\tilde{\text{Y}}-{\tilde{\text{X}}}^{c})\wedge {\tilde{\phi }}^{H}\;dS_{\text{Y}},$$

where ${\tilde{\text{X}}}^{c}$ is the head centroid. Using also the most recent iterates for ${\tilde{\text{U}}}^{H}$ and ${\tilde{\Omega }}^{H}$, the corrections are then given by

A 6 $$\Delta {\text{F}}^{H}={\tilde{\text{F}}}^{H}-{\mathrm{Sp}}^{4}\left(\frac{\mu }{\xi_{⊥}}\right)R^{F}\cdot \left(\begin{array}{c} {\tilde{\text{U}}}^{H}\\ {\tilde{\Omega }}^{H} \end{array}\right)\;\mathrm{and}\;\Delta M^{H}={\tilde{\text{M}}}^{H}-{\mathrm{Sp}}^{4}\left(\frac{\mu }{\xi_{⊥}}\right)R^{M}\cdot \left(\begin{array}{c} {\tilde{\text{U}}}^{H}\\ {\tilde{\Omega }}^{H} \end{array}\right).$$

These corrections appear on the right-hand side of the linear system.
